# Two recently duplicated maize NAC transcription factor paralogs are induced in response to *Colletotrichum graminicola* infection

**DOI:** 10.1186/1471-2229-13-85

**Published:** 2013-05-29

**Authors:** Anna-Maria Voitsik, Steffen Muench, Holger B Deising, Lars M Voll

**Affiliations:** 1 Division of Biochemistry, Friedrich-Alexander-University Erlangen-Nuremberg, Staudtstrasse 5, D-91058, Erlangen, Germany; 2 Faculty of Agricultural and Nutritional Sciences, Phytopathology and Plant Protection, Martin-Luther-University Halle-Wittenberg, Betty-Heimann-Str. 3, 06120, Halle, Saale, Germany

**Keywords:** NAC transcription factor, Maize, *Colletotrichum graminicola*, Biotic stress response, Phylogeny, NAC domain, DNA binding element

## Abstract

**Background:**

NAC transcription factors belong to a large family of plant-specific transcription factors with more than 100 family members in monocot and dicot species. To date, the majority of the studied NAC proteins are involved in the response to abiotic stress, to biotic stress and in the regulation of developmental processes. Maize NAC transcription factors involved in the biotic stress response have not yet been identified.

**Results:**

We have found that two NAC transcription factors, *ZmNAC41* and *ZmNAC100*, are transcriptionally induced both during the initial biotrophic as well as the ensuing necrotrophic colonization of maize leaves by the hemibiotrophic ascomycete fungus *C. graminicola*. *ZmNAC41* transcripts were also induced upon infection with *C. graminicola* mutants that are defective in host penetration, while the induction of *ZmNAC100* did not occur in such interactions. While *ZmNAC41* transcripts accumulated specifically in response to jasmonate (JA), *ZmNAC100* transcripts were also induced by the salicylic acid analog 2,6-dichloroisonicotinic acid (INA).

To assess the phylogenetic relation of *ZmNAC41* and *ZmNAC100*, we studied the family of maize NAC transcription factors based on the recently annotated B73 genome information. We identified 116 maize NAC transcription factor genes that clustered into 12 clades. *ZmNAC41* and *ZmNAC100* both belong to clade G and appear to have arisen by a recent gene duplication event. Including four other defence-related NAC transcription factors of maize and functionally characterized Arabidopsis and rice NAC transcription factors, we observed an enrichment of NAC transcription factors involved in host defense regulation in clade G. *In silico* analyses identified putative binding elements for the defence-induced ERF, Myc2, TGA and WRKY transcription factors in the promoters of four out of the six defence-related maize NAC transcription factors, while one of the analysed maize NAC did not contain any of these potential binding sites.

**Conclusions:**

Our study provides a systematic *in silico* analysis of maize NAC transcription factors in which we propose a nomenclature for maize genes encoding NAC transcription factors, based on their chromosomal position. We have further identified five pathogen-responsive maize NAC transcription factors that harbour putative binding elements for other defence-associated transcription factors in the proximal promoter region, indicating an involvement of the described NACs in the maize defence network. Our phylogenetic analysis has revealed that the majority of the yet described pathogen responsive NAC proteins from all plant species belong to clade G and suggests that they are phylogenetically related.

## Background

NAC transcription factors belong to a large family of plant-specific transcription factors that are expressed in different tissues and at various developmental stages. The founding members of the family, NAM from petunia and ATAF1 and CUC2 from *Arabidopsis*, were described in 1996 and 1997 [1,2], and the initials of these genes were used to derive the name for the newly discovered multigene family. To date, 105 *NAC* genes have been identified in the *Arabidopsis* genome [3], 138 in rice [4], 115 in maize [5], 113 in sorghum, 177 in soybean and 148 in poplar but only around 40 in lower plants like mosses and spike mosses [5,6].

The characteristic feature of this group of transcription factors is the presence of a NAC domain at the N-terminus [2], a stretch of ~160 amino acids highly conserved between the members, which consists of five subdomains A – E [3]. This region serves as a platform for DNA binding, and for homo- or heterodimerizatzion with other NAC proteins [7,8]. Determination of the NAC domain structure revealed a novel transcription factor fold; a twisted β-sheet enclosed by α-helixes [9], which was recently shown to interact with the major groove of the target DNA [8]. The C-terminal region, in contrary, is variable in sequence and length and serves as a transcriptional activator [10,11] or transcriptional repressor [12].

NAC transcription factors regulate a diverse range of processes in plants. The regulatory role of NACs in the development of plant organs like in the shoot apical meristem [2,13], the axillary meristem [14], the cotyledons [1], lateral roots [11,12], the xylem [15,16] or the secondary cell wall [17,18] has been intensively studied. In addition, it has been described that many members of the NAC transcription factor family coordinate the response to abiotic stress. *OsNAC5 and OsNAC6* from rice were shown to be induced by cold, drought and high salinity and to interact with each other and with a third rice NAC transcription factor *SNAC1* to induce the expression of stress-responsive genes. Consequently, rice plants overexpressing *OsNAC5*, *OsNAC6*, *OsNAC10*, *OsNAC45, SNAC1* and *SNAC2* were more resistant to high salt conditions compared to wild type rice plants [19-22]. The expression of *OsNAC63* was also strongly induced in rice roots by high salinity and osmotic stress. *Arabidopsis* plants overexpressing *OsNAC63* exhibited a constitutive upregulation of salinity-inducible genes and produced seeds that were more tolerant to both of these stress conditions [23].

Furthermore, NAC transcription factors are involved in the regulation of senescence in *Arabidopsis*, where overexpression of AtNAP resulted in early senescence of rosette leaves [24], and in wheat, where low transcript levels of *TaNAM* delayed the onset of senescence [25]. In addition, the Arabidopsis NAC transcription factor *RD26* is induced by drought and ABA and plants with reduced *RD26* expression were insensitive to exogenous ABA treatment, indicating a role of RD26 in ABA-signalling [26].

In the past decade, NAC transcription factors were also shown to be involved in the regulation of the plant defence network. For instance, the NAC transcription factor ATAF2 acts as a repressor of *PR* gene expression in *Arabidopsis*[27], while ATAF1 negatively regulates the defence response to necrotrophic fungi and bacterial pathogens [28]. Furthermore, ANAC019 and ANAC055 were involved in the JA-dependent expression of defence genes in Arabidopsis [29]. *OsNAC6* and *OsNAC19* were induced in rice upon challenge with the rice blast fungus *M. grisea*, and the overexpression of *OsNAC6* led to increased resistance towards rice blast [21,30]. Finally, one potato *NAC* gene was induced in leaves after inoculation with *Phytophthora infestans*[31] and *BnNAC1-1, BnNAC5-1 and BnNAC5-7* genes were found to be induced in oilseed rape during flea beetle colonization and *Sclerotinia sclerotiorum* infection [32].

To date, data on the expression profile and possible function of maize NAC transcription factors are limited*. ZmNAM1* (*ZmNAC70* in this report) and *ZmNAM2* (*ZmNAC35*) are expressed in the shoot apical meristem during embryo development, suggesting that they play a similar role as their Arabidopsis and petunia orthologues. Transcripts of *ZmNAC4* were detected in developing endosperm, while *ZmNAC5* and *ZmNAC6*, putative paralogues, were expressed in the coleorhiza [33]. Transcripts of two other NAC transcription factors, *NRP-1* and *Apn-1* were found in the endosperm, the transcript of *Apn-1* was also detected in the developing embryo [34,35]. A group of four NAC transcription factors was shown to be involved in secondary cell wall biosynthesis in maize: *ZmSWN1*, *ZmSWN3*, *ZmSWN6* and *ZmSWN7* were able to complement the phenotype of the Arabidopsis *snd1/ nst1* double mutant, which lacks the secondary cell wall in xylem fibers. Overexpression of each of these four maize NAC transcription factors in *Arabidopsis* wild type led to the ectopic deposition of secondary cell wall, resulting in a curly leaf phenotype similar to that observed for *SND1* overexpressing plants, indicating that *ZmSNWs* are functional orthologues of *SND1*[36].

Although evidence for the involvement of NAC transcription factors in plant defence accumulates, no such data are available for maize yet. Therefore, our aim was to characterize two members of the *NAM* gene family which we found to be induced in maize leaves challenged with *Colletotrichum graminicola*. *C. graminicola* (Cesati) Wilson [teleomorph *Glomerella graminicola* (Politis)] is a causal agent of anthracnose leaf blight and stalk rot, an economically important disease of maize (*Zea mays* L.). The *C. graminicola* infection cycle starts on the leaf surface, where spores germinate. After germination, a specialized infection cell, the appressorium, is differentiated at the tip of the germ tube. The appressorium melanizes and accumulates compatible solutes to develop a high turgor pressure that is subsequently converted into mechanical force to piercing the plant cell wall with the penetration peg. Within the host tissue, the fungus initially produces voluminous primary hyphae that grow biotrophically, i.e. without disrupting the host plasma membrane. This biotrophic phase lasts for approximately 2 days. Subsequently, a switch to necrotrophic growth that involves both a change in lifestyle and hyphal morphology occurs. Spreading of thin, fast growing necrotrophic hyphae, which rapidly colonize and kill the host cells, can be macroscopically observed as extending necrotic lesions. Finally, the pathogen forms acervuli on the surface of the necrotic area, specialized structures mitotically producing conidia, which are distributed to new host tissue by rain splashes [37,38].

In this study, we provide a systematic nomenclature of the maize NAC transcription factor family, which served as the basis to reveal that the two NACs that were induced in the maize – *C. graminicola* interaction and other defense-inducible NAC from maize and other plant species are evolutionary related.

## Results

### Two maize NAC transcription factors are induced in leaves infected with *Colletotrichum graminicola*

**Figure 1:**
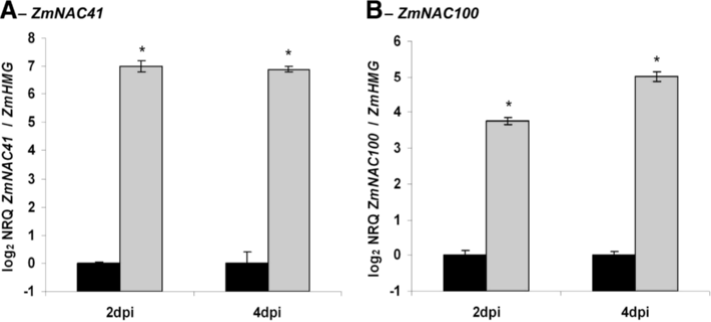
**Induction of maize NAC transcription factors upon infection with *C. graminicola *wild type CgM2.** Relative quantities of *ZmNAC41* and *ZmNAC100* transcripts were analyzed by qRT-PCR and are expressed relative to *ZmHMG* on a log_2_ scale as means ± SE (n = 4). Black bars – mock-treated control leaves, grey bars – infected leaves. Error bars represent the standard error. Asterisks indicate significant differences (P-value < 0.05) to the respective mock control.

**Figure 2:**
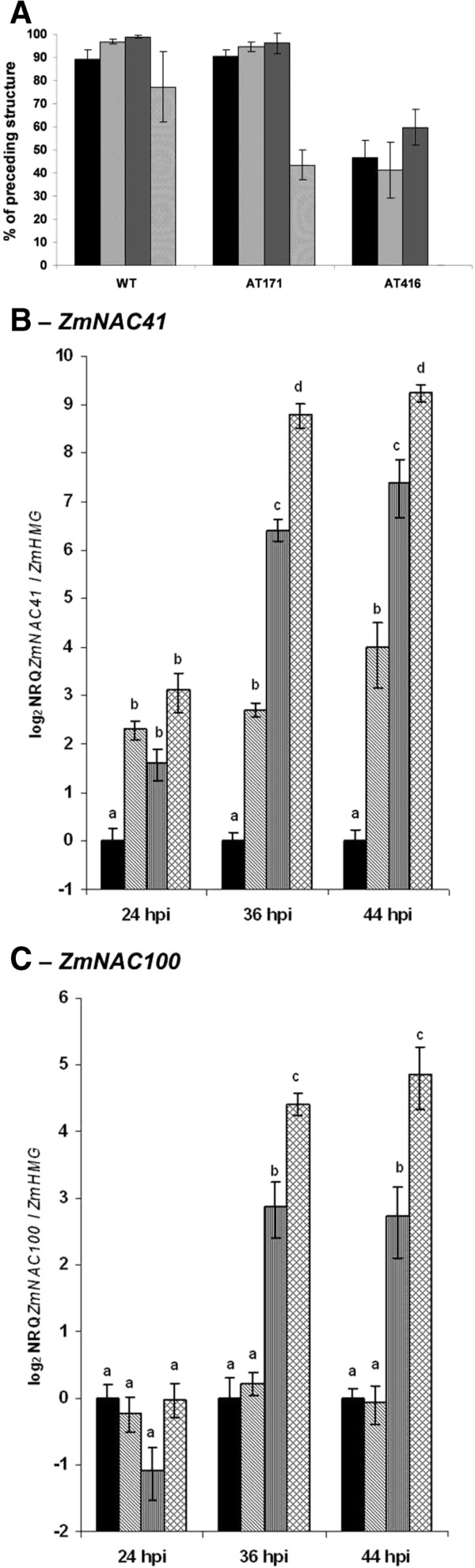
**Induction of*ZmNAC41*and*ZmNAC100*upon infection with*C. graminicola*pathogenicity mutants.** (**A**) Germination rates (black bars), appressoria formation (hatched bars), melanisation rates (vertically hatched bars) and penetration rates (crosshatched bars) of *C. graminicola* CgM2 wild type (left bracket) and the ATMT mutant strains AT171 (middle bracket) and AT416 (right bracket) were assessed at 72 hpi. Four replicate samples per genotype with approx. 100 conidia were assessed and are given ± SE. The data for every developmental stage is given in percent relative to the total number of infection events that exhibited the preceding developmental stage. (**B**) and (**C**) Relative quantities of *ZmNAC41* (**B**) and *ZmNAC100* (**C**) transcripts were analyzed by qRT-PCR and are expressed relative to *ZmHMG* on a log_2_ scale as means ± SE (n = 4). Mock treated leaves - black bars, wild type strain CgM2 - cross-hatched bars, AT416 mutant - diagonal hatched bars, AT171 mutant - vertical hatched bars. Dissimilar letters indicate significant differences (P-value < 0.05) between the treatments.

In order to investigate which host genes respond to *C. graminicola* infection at the different stages of the interaction, we compared the transcriptome of leaves that were spray-inoculated with 2 × 10^6^ conidia/ml to mock-treated control leaves during the biotrophic phase at 36 hpi and after the switch to the necrotrophic phase at 96 hpi by microarray analysis (see [39]). At 36 hpi, more than 313 genes were differentially regulated (fold change > 2), of which 251 were upregulated in infected leaves. In this set, two genes encoding the putative NAC transcription factors *ZmNAC41* and *ZmNAC100* were found, which were also induced during the necrotrophic leaf colonization at 96 hpi. To confirm the microarray data, transcript levels of both *NAC* genes were assessed at 2 and 4 dpi by qRT-PCR (Figure 1). While *ZmNAC100* transcripts were induced 4–5 fold, *ZmNAC41* was induced 7-fold. As spray-inoculation only led to infection of a fraction of the epidermal cells, the induction of both *NACs* transcripts is likely significantly higher in the infected cells. To determine the induction kinetics at earlier time points of the interaction, we assessed *Zm*NAC41 and *Zm*NAC100 transcript amounts in dip-inoculated leaves, where the proportion of infected tissue is higher compared to spray-inoculated leaves (see Methods section). We employed both *C. graminicola* wild type (WT) strain CgM2 and mutant strains generated by *Agrobacterium tumefaciens*-mediated transformation (ATMT), which are affected in virulence to different extent. While fungal penetration was reduced by 50% in mutant AT171, which is weakly affected in virulence (see [40]), mutant AT416 was unable to efficiently penetrate host tissue and was strongly affected in virulence (Figure 2A). In WT-infected leaves, *ZmNAC41* was weakly induced already at the pre-penetration stage at 24 hpi, but massive transcript accumulation coincided with the time of the establishment of biotrophy at 36 hpi (Figure 2B). *ZmNAC41* was also induced in the interactions with the two mutants at all tested time points and the expression level was positively correlated with the virulence of the employed strain (Figure 2B). In contrast, the expression of *ZmNAC100* was first induced after successful penetration of the WT and the mutant AT171 strain into the host tissue at 36 hpi. In contrast, mutant strain AT416 failed to induce the *ZmNAC100* gene (Figure 2C). The timing of infection was confirmed by microscopic observation of the infected leaves (data not shown). Our data demonstrate that *ZmNAC100* is induced only upon successful penetration of *C. graminicola* into the host tissue, which suggests that this NAC could be a part of the induced defence response. Correlation of the expression level of both *NACs* genes with fungal virulence suggests that they could be a potential compatibility factors in the interaction of maize with *C. graminicola*.

### *ZmNAC41* and *ZmNAC100* are induced by defence signals and during leaf senescence

**Figure 3:**
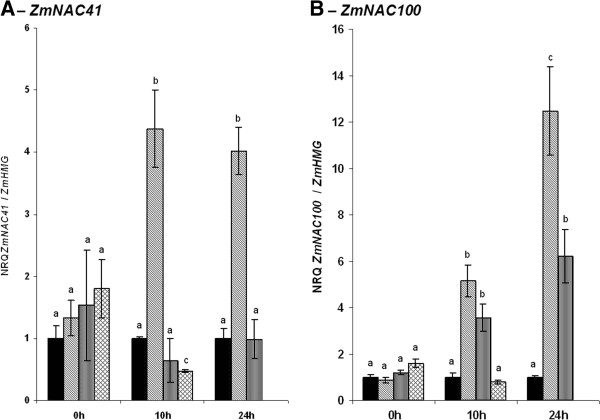
**Induction of*ZmNAC41*and*ZmNAC100*in response to hormone and hormone analog treatments.** Relative quantities of *ZmNAC41* (**A**) and *ZmNAC100* (**B**) transcripts in mock-treated control leaves (black bars) and after 0 h (left bracket), 10 h (middle bracket) and 24 h (right bracket) of treatment with 1 mM JA (diagonal hatched bars) or 1.3 mM INA (vertical hatched bars) and after 0 h and 10 h of treatment with 5 mM ACC (cross-hatched bars) were analyzed by qRT-PCR and are expressed relative to *ZmHMG* on linear scale as means ± SE (n = 4). Dissimilar letters indicate significant differences (P-value < 0.05) between the treatments.

As both *ZmNAC41* and *ZmNAC100* responded to biotic stress, we assessed their responsiveness to phytohormones involved in coordinating plant defence response and treated maize leaves with jasmonic acid or 2,6-dichloroisonicotinic acid (INA), an analogue of salicylic acid, or the ethylene precursor 1-aminocyclopropane-1-carboxylic-acid (ACC), a precursor of ethylene. Both transcription factors were induced by jasmonic acid already 10 hours after treatment (hat), and transcripts of *ZmNAC100* accumulated further up to 24 hat (Figure 3). Moreover, transcript accumulation of *ZmNAC100*, but not that of *ZmNAC41*, was enhanced by exogenously applied INA. These results suggest that *ZmNAC41* is specifically induced by JA, and neither *ZmNAC41* nor *ZmNAC100* responded to ethylene (Figure 3). However, the induction of *ZmNAC41* and *ZmNAC100* during the compatible interaction with *C. graminicola* was approx. 100-fold higher as compared to the induction by JA and INA (Figure 3).

**Figure 4:**
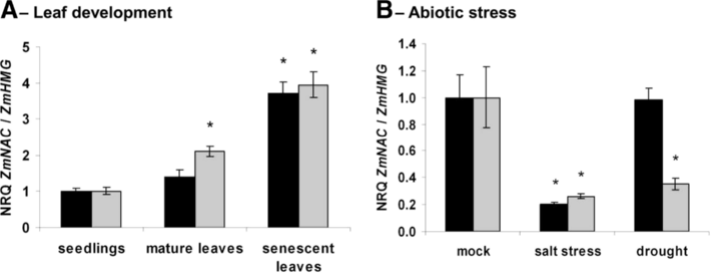
**Transcript amounts of*ZmNAC41*and*ZmNAC100*during leaf development and in response to abiotic stress treatments.** Relative quantities of *ZmNAC41* (black bars) and *ZmNAC100* (grey bars) were analyzed at different stages of the leaf development (**A**) and in leaves upon drought or high salinity conditions (**B**), as indicated below the graphs. Relative transcript amounts were determined by qRT-PCR and are expressed relative to *ZmHMG* on linear scale as means ± SE (n = 4). Asterisks indicate significant differences (P-value < 0.05) of the seedlings to the mature and senescent leaves (**A**) or stress-treated leaves to the respective mock-treated control (**B**).

Many NAC transcription factors are involved in gene regulation during the senescence program (reviewed by [41,42]), during which defense-related genes are also induced. Transcript levels of both, *ZmNAC41* and *ZmNAC100* increased during leaf development and were about 4-fold greater in senescent leaves, as compared to seedlings (Figure 4A).

### *ZmNAC41* and *ZmNAC100* are downregulated during salt stress

Some members of the NAC transcription factor family, such as *OsNAC6*, were described to have overlapping roles in response to both biotic and abiotic stresses [21]. Therefore, we have subjected maize plants to drought or high salinity conditions and evaluated the transcript level of the two *NACs* genes. Both transcription factors were down-regulated during salt stress and the transcripts of *ZmNAC100* also declined during drought stress (Figure 4B). These results demonstrate that both *ZmNAC41* and *ZmNAC100* are distinctly regulated in biotic and abiotic stress conditions.

### Additional maize NAC transcription factors are induced during the defense response

We further analysed, whether other maize NACs are also associated with the defence response. As shown by our transcriptome analysis, *ZmNAC15* and *ZmNAC97* were weakly induced during the necrotrophic stage of *C. graminicola* infection (Table 1). From the four NAC genes induced in the *C. graminicola* maize interaction, only *ZmNAC41* was also upregulated in response to the fungal biotroph *Ustilago maydis*[43]. However, the induction of *ZmNAC41* by *U. maydis* has only been observed at 12 hpi, prior to active defense suppression by the smut fungus [43]. In addition, two other NACs, *ZmNAC36* and *ZmNAC38* were transcriptionally repressed in the interaction with *U. maydis* upon tumor formation at 4 dpi.

**Table 1:** **Maize NAC transcription factors differentially regulated in the interaction with *C. graminicola *and *U. maydis***

	***Colletotrichum graminicola***				***Ustilago maydis***					
**NAC gene**	**36 hpi**		**96 hpi**		**12 hpi**		**96 hpi**		**192 hpi**	
	**f.c.**	p-val	**f.c.**	p-val	**f.c.**	p-val	**f.c.**	p-val	**f.c.**	p-val
ZmNAC100	**3.3**	0.19	**204**	2.8 ∙ 10^-6^						
ZmNAC41	**3.4**	0.02	**45**	3.5 ∙ 10^-4^	**7.0**	0.08				
ZmNAC15			**3.2**	0.05						
ZmNAC97			**2.2**	0.006						
ZmNAC38					**3.7**	0.09			**−2.7**	0.003
ZmNAC36							**−2.4**	0.07		

Linear fold change values (f.c.) of gene expression are given; negative values represent a down regulation. Only fold changes > 2 are displayed. Data were extracted from the microarray analysis of the maize transcriptome [39] and p-values (p-val) were calculated using bioconductor (http://www.bioconductor.org).

To identify the regulatory circuitry behind the observed regulation of NAC genes in the two pathosystems, we have scrutinized the upstream promoter regions of the identified maize NAC genes for the presence of binding motifs for defence-associated transcription factors to elucidate if certain promoter elements could confer the specific response towards *C. graminicola* or *U. maydis*. All promoters contained a core NAC transcription factor binding site that had been predicted from the promoter element analysis of *ANAC019* and *ANAC092*[7]. The entire NAC-binding motifs identified for the two Arabidopsis NACs could be found in all analyzed promoters in one to five copies, suggesting that other NAC proteins could bind to the promotors of the analysed NACs as homo- or heterodimers (Table 2). Furthermore, the promoters of all except *ZmNAC97*, contained binding sites for ERF and TGA transcription factors, which regulate the expression of target genes in response to ethylene or salicylic acid, respectively. A Myc2 binding site, present in the promoters of many jasmonic-acid responsive genes, was found in *ZmNAC15*, *ZmNAC38* and *ZmNAC41*, while a WRKY-binding motif could be detected in *ZmNAC15*, *ZmNAC36, ZmNAC41* and *ZmNAC100*. Despite considerable conservation of ERF, TGA, Myc2 and WRKY binding motifs, the promoters of the six analysed NAC genes differ in their individual motif composition. In the proximal region 500 bp upstream of the start codon, putative ERF binding motifs were only present in *ZmNAC15* and *ZmNAC38*, while all potential WRKY binding sites were located in this proximal region. Interestingly, a *Whirly*-binding motif was found only in *ZmNAC41* and *ZmNAC100*, the only members induced during the early interaction of maize with *C. graminicola*. In summary, the *ZmNAC15, ZmNAC36, ZmNAC38, ZmNAC41* and *ZmNAC100* genes all contained potential binding elements for other transcription factors known to be involved in the plant defence network within the proximal promoter region.

**Table 2:** **Binding motifs of defence-associated transcription factors present in the promoter elements of maize NAC transcription factors upregulated in response to *C. graminicola *and *U. maydis***

**NAC gene**	**Myc2**	**Whirly**	**ERF**	**WRKY**	**TGA**	**NAC**
***ZmNAC100***		−582 bp (GTCAAAA)	−691 bp (GCCGCC)	**−464 bp (TTGACC)**	−556 bp (TGACG)	−833 bp (AGACGTG)
		−1737 bp (GTCAAAT)			- 1471 bp (TGACG)	−920 bp (TGTCGTG)
						−963 bp (ATGCGTG)
***ZmNAC41***	−1284 bp (CATGTG)	−1155 bp (GTCAAAT)		**−348 bp (TTGACC)**		−1166 bp (TAGCGTGAT)
						−1615 bp (TAACGTATA)
***ZmNAC15***	−795 bp (CATGTG)		**−364 bp (AGCCGCC)**	**−349 bp (TTGACC)**	−530 bp (TGACG)	−906 bp (TTGCGTA)
	−1445 bp (CATGTG)		- 592 bp (GCCGCC)			−945 bp (TTTCGTA)
			−1333 bp (GCCGCC)			−1023 bp (AGCCGTA)
			−1464 bp (GCCGCC)			−1319 bp (TTGCGTG)
			−1566 bp (GCCGCC)			
			−1569 bp (GCCGCC)			
***ZmNAC97***						−1718 bp (TTACGTG)
						−1825 bp (TTGCGTG)
***ZmNAC38***	−1604 bp (CATGTG)		**−351 bp (GCCGCC)**		−857 bp (TGACG)	**−254 bp (TGACGTA)**
			−**369 bp (GCCGCC)**		−952 bp (TGACG)	−**399 bp (ATCCGTA)**
			−**384 bp (GCCGCC)**		−1101 bp (TGACG)	−915 bp (ATTCGTA)
			−**482 bp (GCCGCC)**			−1051 bp (AGGCGTG)
						−1106 bp (AGGCGTG)
***ZmNAC36***			−1343 bp (GCCGCC)	−510 bp (TTGACC)	−1153 bp (TGACG)	−761 bp (TGGCGTG)
			−1346 bp (GCCGCC)		−1211 bp (TGACG)	

The distance in base pairs from the respective start codon and the sequence of each element was given.

### Analysis of the family of maize NAC transcription factors

The fact that promoter elements were quite conserved between the six analysed NAC transcription factors prompted us to explore their evolutionary relation. Using the unassembled maize genome information, Shen et al. [6] identified 177 putative maize *NAC* genes. Since an assembly of the B73 maize reference genome became available [44], we analyzed the NAC transcription factor family based on the assembled B73 genome information.

**Figure 5:**
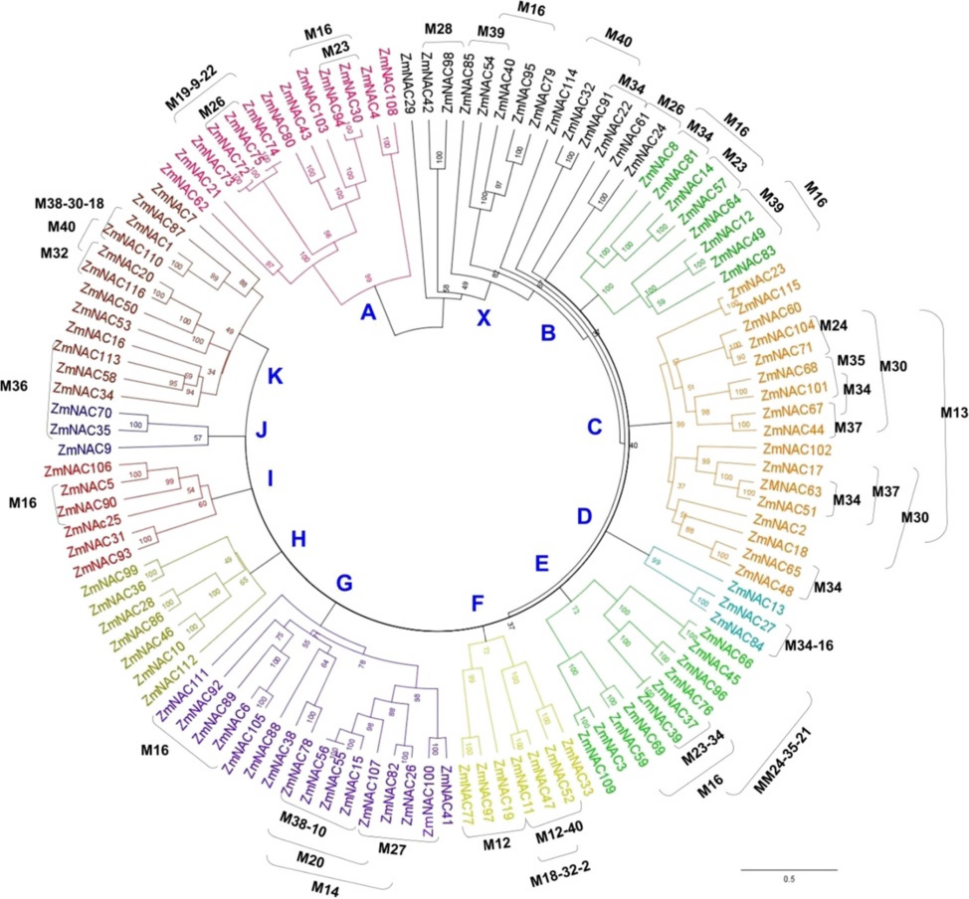
**Phylogenetic analysis of maize NAC proteins.** The 116 maize NAC proteins were extracted from the published B73 genome sequence as described in the text and are numbered according to position in the genome (starting with the short arm of chromosome I, see Additional file 1: Table S1). Bootstrap values are indicated at the nodes, the bar at the bottom of the figure depicts the distance scale for branch length. Proteins with identical C-terminal motifs were labelled with the brackets and the respective motif’s number was given (see Additional file 3: Table S2).

We employed the conserved NAC domain of *ZmNAC41* and *ZmNAC100* as a query to search against the peptide database (release 5b.60) deposited at http://maizesequence.org. Moreover, gene models for putative maize NAC transcription factors, deposited at Grassius Grass Regulatory Information Server (http://www.grassius.org/index.html), were blasted against the assembled maize genome. As an outcome of both surveys, 116 putative maize NAC genes (excluding alternative splice variants) have been identified, which were renamed using the acronym ZmNAC and ascending Arabic numbers due to chromosomal localisation as a suffix (starting with the short arm of chromosome 1, see Additional file 1: Table S1). Multiple alignment performed on the whole set of putative NAC protein sequences served for the construction of a phylogenetic tree, which revealed that the family can be divided into 12 clades (Figure 5). Phylogenetic trees generated from the entire NAC sequences (Figure 5) were very similar to those obtained from an alignment of the NAC domains only (not shown), indicating that the NAC domains allow for most of the distinction between individual clades.

**Figure 6:**
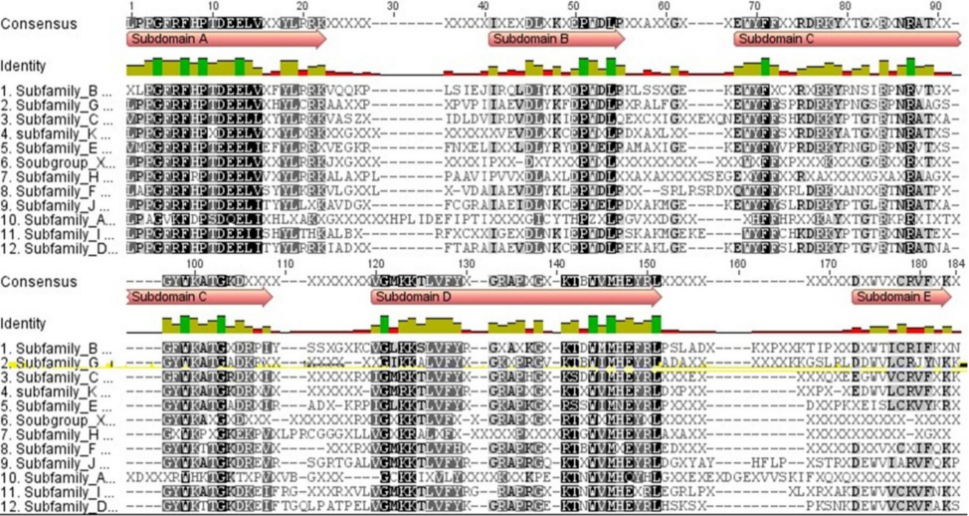
**Architecture of maize NAC domains.** A multiple alignment of the consensus sequences of the NAC domains from each clade was compiled using ClustalW 2.0. Amino acid residues present in at least 50% of the subfamiliy members are displayed in the consensus sequences. For a complete alignment, please see Additional file 2: Figure S1.

**Figure 7:**
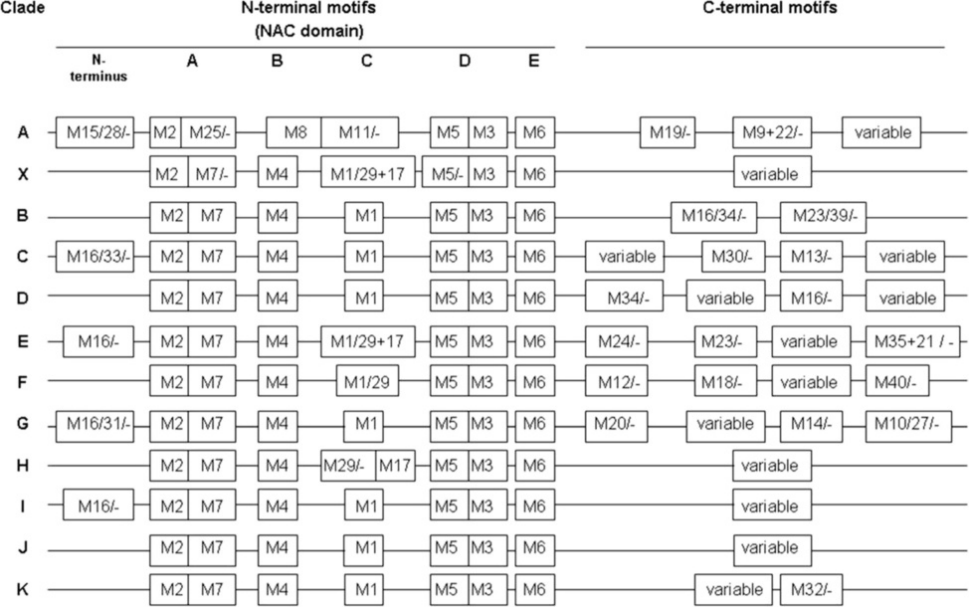
**Positions of the motifs detected within the maize NAC proteins.** The motifs detected with MEME (Multiple Em for Motif Elicitation, http://meme.nbcr.net/meme/cgi-bin/meme.cgi) are marked. For a complete list of motifs, please see Additional file 3: Table S2. For the simplicity reasons, all proteins are aligned to one length. The term “variable” is used if more than three variants of the sequence are present at the respective spot, the absence of the given motif is marked with a dash.

**Figure 8:**
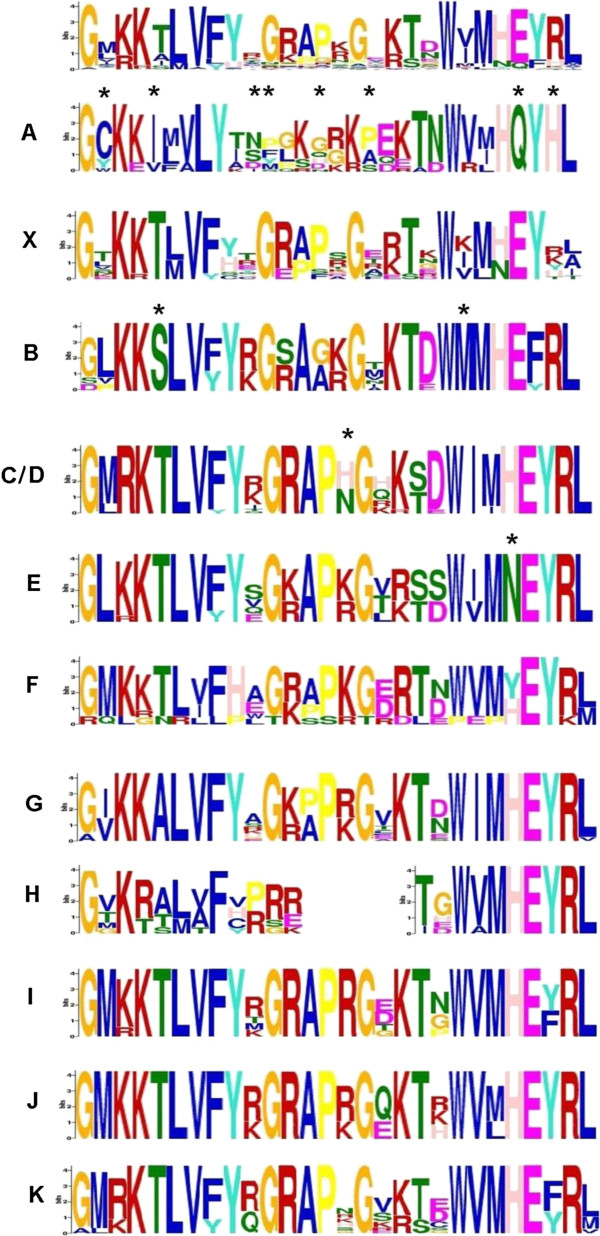
**Divergence of the NAC subdomain D consensus sequence.** Comparison of the subdomain D aa consensus sequences from individual NAC clades after analysis with MEME Multiple Em for Motif Elicitation. At the top, the consensus aa sequence for subdomain D of all 116 NAC sequences is given and below consensus sequences for the indicated clades are given according to the letter left to the motifs. For clade H, the middle part of subdomain D consists of a stretch of variable amino acid residues Amino acid residues specific for individual clades are highlighted by asterisks.

An alignment of the consensus sequences generated for each clade revealed the typical domain architecture of the NAC proteins. The N-terminal part of the proteins, which includes the NAC domain, was well conserved between the clades, while the C-terminal region was highly divergent even between the members of the same clade (Additional file 2: Figure S1). As described for the Arabidospis and rice NAC transcription factor families [3,4] and in two surveys using genomic information from 9 and 11 different plant species [5,6], respectively, five highly conserved subdomains A-E, separated by about 10–20 aa, have been distinguished in the NAC domain (Figure 6). To identify consensus sequences of the subdomains A-E for all clades, the NAC domains of all maize NAC transcription factors were screened with MEME (http://meme.sdsc.edu/meme/cgi-bin/meme.cgi, [45]). All clades except clade A shared common motifs (cut off p-value 1∙e^-10^) within the NAC domain (Figure 7). More detailed analysis of these common motifs revealed that the conservation of each motif was higher within the same clade than in the comparison to other clades. As shown for subdomain D (Figure 8), single amino acid residues differed between the individual NAC clades, except for clades C and D, which cannot be distinguished by subdomain D. A high similarity between the NAC subdomains A, D and E was evident between clade A and the other clades, while the NAC subdomains B and C were divergent. Some NAC proteins contained additional motifs in front of the NAC domain. For instance, four members of the clade C shared one leading motif, while another leading motif was present in six members of the clade G and six NACs from other clades.

In the C-terminal part of the protein sequences, in total twenty four distinct motifs (cut off p-value 1∙e^-10^) have been identified (Figure 7 and Additional file 3: Table S2), some of which were specific to certain clades and subclades as described in the following paragraph. The C-terminus of clade F was distinct from all other clades. First, 6 of 7 clade members contained the QYGAPF motif (motif 12), which is also present in six rice NACs (representing motif 39 in [4]), and in addition, two different kinds of long C-terminal extensions were present in these members. Furthermore, subfamiliy G has even been divided into three subclades, based on the presence of motif SYDDIQ (motif 10) in subclade G1 and of motif NLDDLQ (motif 27) in subclade G2, which were both absent from the C-terminus of the third subclade. Similarly, one subclade of clade E consisted of six members that all carried a long C-terminal extension that contained three different motifs: ARS (motif 24), IDELS (motif 35) and KIWDWNP (motif 21). Furthermore, 11 members of four different subclades from clade C contained motif TDW (motif 13) and LPLE (motif 30). The latter motif is also present in the C-terminal domains of Arabidopsis and rice NACs (and corresponds to motif iii in [3]). Finally, the 50 aa motif MAAESNL (motif 9) was specific for four members of clade A, which share between 83% (ZmNAC73 vs. ZmNAC75) and 99% (ZmNAC74 vs. ZmNAC75) homology and appear to be the result of recent duplications.

However, some motifs were shared between members of different clades. Motif 36 (CFS) was present in five members of clades K and J and in some Arabidopsis and rice NACs (representing motif ix/x in [3]). Furthermore, motif EGSPT (motif 40) was common to members of clades F and K. Motif QT (motif 23) was shared between clades A, B and E, while motif HH/QHH (motif 34) was common to members of clade X, B, C, D and E. The HH/QHH motif is also present in four rice NACs (representing motif 31 in [4]). Some members of clade X contained motifs that were also found in clade A and B, respectively, which reflects the phylogenetic position of clade X between A and B.

### Clade G is enriched in defence-associated NAC transcription factors

**Figure 9:**
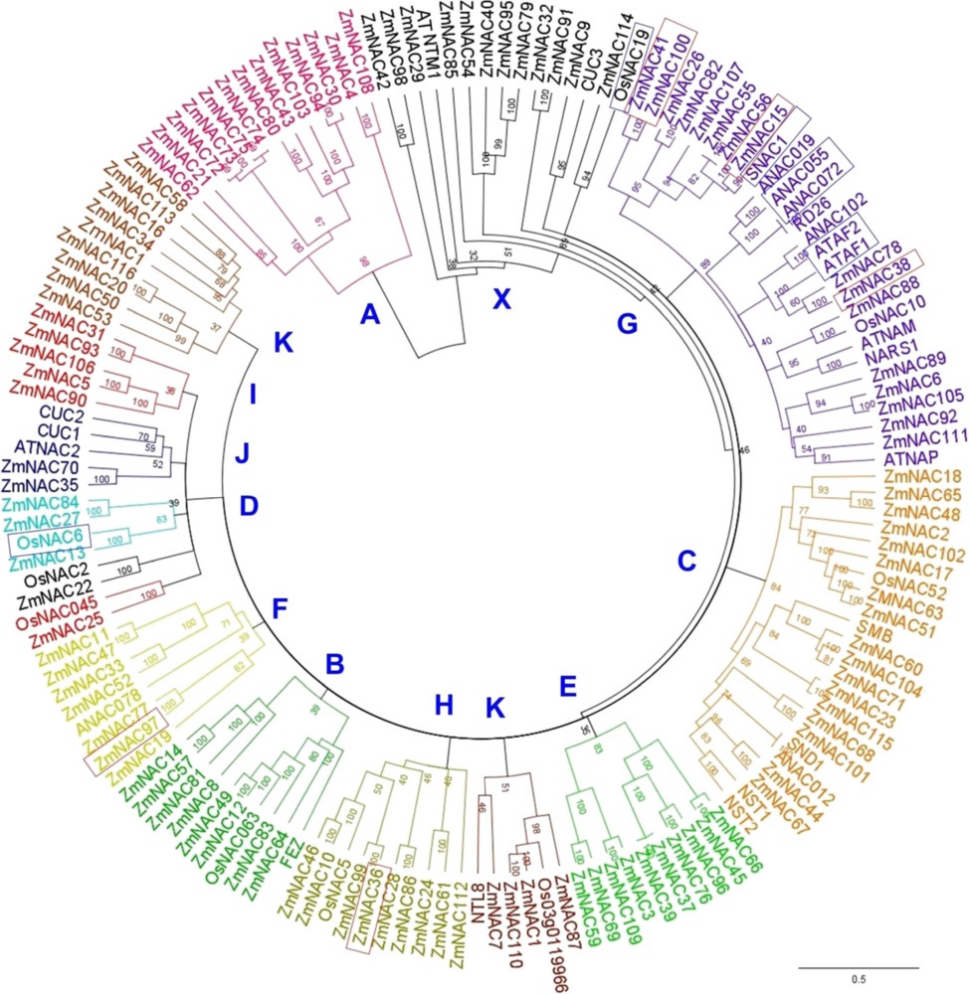
**Phylogenetic relationship of NAC proteins associated with the plant defence response.** In comparison to the pedigree shown in Figure 5, all Arabidopsis and rice NAC proteins with known function were included. Defence-associated NAC proteins are labeled with red (maize) and blue (Arabidopsis, rice) boxes. Clade G was enclosed by dashed blue lines. Bootstrap values are indicated at the nodes, the bar depicts the distance scale for branch length.

Protein sequence comparison showed that *ZmNAC41* and *ZmNAC100* are closely related; sharing 78% similarity of the whole sequence and 87% in the NAC domain. Thus, these two transcription factors belong to clade G, as revealed by a phylogenetic analysis (Figure 5). Checking the gene duplication data available for maize [44] further revealed that the two NACs have arisen from segmental duplication between long arms of chromosome 3 (*NAC41*) and chromosome 8 (*NAC100*). We were interested to know if the other maize NACs that were associated with defence responses towards the fungal pathogens *C. graminicola* and *U. maydis* (see Table 1) are related to *ZmNAC41* and *ZmNAC100*. Phylogenetic analysis revealed that *ZmNAC15* and *ZmNAC38* were also members of clade G, while *ZmNAC36* and *ZmNAC97* were divergent from all of the other five proteins, respectively (Figure 5). Including all functionally characterized Arabidopsis and rice NACs to the phylogenetic tree of maize NACs, we found that four Arabidopsis defence-associated NACs also clustered into clade G (Figure 9). Arabidospis ATAF1 was reported to be involved in the defence response against bacterial pathogens and necrotrophic fungi [28], while the closely related ATAF2 was shown to regulate the expression of *PR* genes [27]. ANAC019 and ANAC055 were described to be involed in the regulation of the JA-dependent defence response [29]. However, the two only rice NACs that are known to be involved in the response towards biotic stress, *OsNAC6* and *OsNAC19*, were found outside clade G (Figure 8). In summary, eight out of twelve defence-associated NACs from maize, rice and Arabidopsis are members of clade G, while the four other were clustering to the separate families. As of our current knowledge, clade G seems to be enriched in transcription factors involved in response to biotic stress, suggesting that an ancestral NAC of clade G might have acquired its role in defence regulation earlier than NAC proteins from different clades of the family. However, this interpretation is limited by the functional characterization of orthologs of the relevant NAC clades.

## Discussion

### The involvement of NAC proteins in the plant defence response network

In this study we have characterised two maize NAC transcription factors; *ZmNAC41* and *ZmNAC100*, which are induced during the interaction of maize with *C. graminicola*. The accumulation of *ZmNAC41* transcript preceeded fungal penetration of the host tissue, suggesting that this transcription factor is activated as a part of the basal defence response. A similar induction pattern was described for the *HvNAC6* from barley [46], which was transcriptionally induced in epidermal cells shortly after inoculation with *Blumeria graminis* f. sp. *hordei* (Bgh). Silencing of *HvNAC6* reduced penetration resistance and the number of papilla formed in response to fungal penetration [46]. A deletion of *ATAF1* in Arabidopsis, an *HvNAC6* orthologue, compromised non-host resistance to Bgh, which was shown to be predominantly associated with papilla formation Jensen et al. [46]. Based on these observations, the *ZmNAC41*, *HvNAC6* and *ATAF1* orthologs are hypothesized to integrate the early transcriptional events upon PAMP recognition during the basal defence response.

However, the highest accumulation of *ZmNAC41* was reached upon successful penetration of *C. graminicola* into the maize tissue, while the transcription of the other maize NAC transcription factor, *ZmNAC100*, was exclusively induced during the post-penetration stage of the infection. These data further suggest that both transcription factors described in this study are also associated with induced defence responses at later stages of infection. Induced defence reactions are controlled by phytohormones like jasmonic acid, salicylic acid and ethylene. We have revealed that both maize NAC transcription factors described here are responsive to jasmonic acid, while transcription of *ZmNAC100* was also enhanced by salicylic acid, indicating that both transcription factors are indeed involved in phytohormone triggered defence responses. Similarly, it was shown that *OsNAC5* and *OsNAC6* from rice are strongly induced by methyl jasmonate, although transcripts of both genes accumulated to a similar level as during drought and cold stress [22]. Two Arabidopsis genes coding for NAC transcription factors, *ANAC019* and *ANAC055* were also responsive to methyl jasmonate, in a COI1- and AtMYC2-dependent manner. Moreover, studies with the *anac019/ anac055* double knock-out and overexpression lines revealed that the expression of other JA-responsive genes, like *VEGETATIVE STORAGE PROTEIN 1* (*VSP1*) and *LIPOXYGENASE2* (*LOX2*), is regulated by ANAC019 and ANAC055 [29], suggesting that these two NAC transcription factors are part of a JA feedback loop. The promoter element analysis of six pathogen induced maize NAC transcription factors in our study has revealed response elements for ERF, WRKY, TGA and NAC transcription factors within 500 bp upstream of the ATG in five of the six analysed genes, suggesting an involvement of these five NACs in the transcriptional network controlling the plant defence response.

### Most NAC transcription factors involved in plant defence are phylogenetically related

Interestingly, *ATAF1, HvNAC6, ANAC055*, *ZmNAC41* and *ZmNAC100* as well as the two other pathogen inducible maize NAC transcription factors *ZmNAC15* and *ZmNAC38* belong to NAC clade G. Taking our data and the recent analyses of rice NACs by Nuruzzaman et al. [47] and Zhu et al. [5] into account, almost two thirds of all studied defence-induced NAC transcription factors belong to clade G. Interestingly, this clade is one of three evolutionary ancient subclades and contains most of the moss and lycophyte NAC representatives analysed [5]. *Physcomitrella patens*, the most ancient species harboring NAC transcription factors, possesses genes of the oxylipin pathway like allene oxide synthase (AOS, [48]), allene oxide cyclase (AOC, [49]) and lipooxygenase (LOX, [50]). However, jasmonates have not yet been detected in this moss. Nevertheless, it appears tempting to speculate that one of the first acquired functions of NAC transcription factors might have been the perception of oxylipins since more than 410 million years ago, a time estimate, which is based on the analyses by Zhu et al. [5].

The comprehensive phylogenetic analysis of 837 NAC transcription factor genes from 9 fully sequenced species of diverse evolutionary position by Zhu et al. [5] has revealed 21 NAC clades, of which 15 contain maize orthologs. In contrast, our analysis has revealed 12 NAC clades. If we take into account that clade C in our analysis can be divided into two subclades and clade G can be divided into three subclades, our analysis has generated an equal number of discernible clades compared to Zhu et al. [5]. However, the number of clades described in the analysis of Arabidopsis and rice NAC transcription factors [3,4,6,51] deviates from study to study. This indicates that the diversity of the employed genome information determines the computation of NAC clades due to the available number of protein sequences.

## Conclusion

In this study, we have identified six maize NAC transcription factors that are induced upon challenge by fungal attack and *in silico* analysis revealed the presence of promoter elements that supports an involvement of five maize NACs in the defence transcription network. The two members that responded strongly to penetration by *C. graminicola* and that were studied in more detail, *ZmNAC41* and *ZmNAC100*, were predominantly JA responsive. We have generated a systematic classification of maize NAC genes. On the basis of our phylogenetic analysis, we could reveal that the majority of those NAC transcription factors that have yet been described to be involved in the defence network of higher plants are monophyletic.

In summary, our study adds to a number of previous reports on the involvement of NAC transcription factors in the Arabidopsis, rice and barley defence response. Thus, an increasingly large number of NAC transcription factors seems to be involved not only in the regulation of developmental processes and abiotic stress responses, but also in the regulation of biotic stress responses.

## Methods

### Cultivation of plant and fungal material

Maize plants (*Zea mays* L.) cv. Nathan were cultivated in phytochambers at a PFD of 400 μE ∙ m^-2^ ∙ s^-1^ in a 14 h/10 h light/dark cycle as described by and *Colletotrichum graminicola* (Ces.) Wils. [teleomorph *Glomerella graminicola* (Politis)] was grown as described in [40].

### Infection assays

For the infection experiments *Colletotrichum graminicola* wild type isolate CgM2 of *C. graminicola* and ATMT-generated pathogenicity mutants [40] were used. Spores of *C. graminicola* were washed off from 2 weeks old OMA plates with 1 ml distilled water and diluted to a final concentration of 2 × 10^4^ (low titer) or 2 × 10^6^ (high titer) spores/ml. As specifically stated in the text, fully expanded fourth leaves of two weeks old maize plants were either sprayed with a spore suspension of a high titer (2 × 10^6 ^spores/ml), containing additionally 0.02% (v/v) Tween-20 or dipped in a spore suspension of a low titer (2 × 10^4^ spores / ml) for 24 h. Sprayed plants were kept in 100% relative humidity conditions for the next 24 h. Mock-treated leaves were sprayed with 0.02% (v/v) Tween-20 in Milli-Q distilled water or dipped in pure distilled water, respectively. As evaluated by microscopy of acid fuchsin stained leaf material [40], fungal proliferation was comparable in dip-inoculated and in spray inoculated leaves, although the conidia titer was 100-fold lower in dip-inoculated material. However, dip-inoculation resulted in a much more homogenous infection of the treated leaf segments. Leaves were collected at 24, 36 or 44 h after inoculation, frozen immediately in liquid nitrogen and subjected to further analysis.

### Hormone treatment

Hormone treatments were performed with 1 mM jasmonic acid (JA), 1.3 mM of the SA analog 2,6-dichloroisonicotinic acid (INA), and 5 mM of the ethylene precursor 1-aminocyclopropane-1-carboxylic-acid (ACC). Fourth leaves of two weeks old maize plants were cut submerged in distilled water and incubated in 15 ml 0.2% ethanol containing the indicated hormone concentrations or no addition for mock controls. Leaves were collected after 0, 10 or 24 h of treatment, frozen immediately in liquid nitrogen and subjected for RNA extraction.

### Abiotic stress assay

Maize plants were grown with regular watering to 100% field capacity ever other day. Three weeks old plants were subjected to drought and high salinity by withholding water or continuing irrigation with 200 ml of 200 mM sodium chloride. Mock-treated plants were watered as before. After one week of stress treatment, all leaves of each plant were harvested, pooled and subjected for RNA extraction.

### RNA extraction

Frozen plant material was ground with mortar and pestle in liquid nitrogen to a fine powder and extracted according to the method described by Chomczynski and Sacchi [52].

### qRT-PCR

1 μg of total RNA was treated with DNase I (Fermentas, St. Leon-Rot, Germany) and RT reaction was performed with Revert Aid™ H Minus Reverse Transcriptase (Fermentas) in total volume of 40 μl according to the manufacturer’s protocol. qRT-PCRs was performed with 1 μl of cDNA from the above RT reactions using 2 × Brilliant II SYBR® Green QPCR Master Mix (Stratagene, Waldbronn, Germany) and 200 nM of upstream and downstream primer each in total volume of 20 μl. The reactions were run on Mx3000P™ System and analyzed with MxPro QPCR Software (Stratagene). Relative transcript amounts of *ZmNAC41* were evaluated with forward primer 5′-GATGAAGATGAGTGTCCACGAT-3′ and reverse primer 5′-CCAACCACATACGTATTATCTAACG- 3′ (product size- 149 bp), for *ZmNAC100* forward primer 5′-TCTGAGAGTTGCTGTGATGGAA-3′ and reverse primer 5′-TAACCCTTACAAGACTACCAGCAAC-3′ were used (product size – 134 bp). The expression level of both NACs was normalized to transcript level of *ZmHMG* gene, evaluated with forward primer 5′-GCTTGGTCTCCATGCTTCATCTAA-3′ and reverse primer 5′-CGGTGAAACTGAACTGAACACAAC-3′, giving the 130 bp product. Target gene transcript amounts were normalized to *ZmHMG* and were calibrated to reference samples as indicated in the respective figure legends.

### Microarray analysis

For transcript profiling, the microarray data described by Voll et al. [39] were employed.

### Phylogenetic analyses

Maize sequences were downloaded from http://www.maizesequence.org (Release 5b.60) and from the Grassius Grass Regulatory Information Server (http://www.grassius.org/index.html). *Arabidopsis* sequences were obtained from the Arabidopsis Information Resource (TAIR) (http://www.arabidopsis.org/) and rice sequences were downloaded from GreenPhylDB (http://greenphyl.cirad.fr/v1/cgi-bin/greenphyl.cgi).

Multiple alignments of protein sequences were performed with the program ClustalW 2.0 [53] and phylogenetic trees were build using the UPGMA method implemented in the program Geneious Pro 5.4.3 [54] with 100 replicates for bootstrap assessment. Protein sequences were screened for common motifs with MEME Multiple Em for Motif Elicitation (http://meme.sdsc.edu/meme/cgi-bin/meme.cgi) [45].

### Statistical analyses

All statistical analyses were performed with a two-tailed, unpaired Student’s t test (P < 0.05)

## Abbreviations

ABA: Abscisic acid; ACC: 1-aminocyclopropane-1-carboxylic-acid; At: *Arabidopsis thaliana*; ATMT: *Agrobacterium tumefaciens* mediated transformation; ERF: Ethylene response factor; ET: Ethylene; hpi: hours post infection; hpt: Hours post treatment; INA: 2,6-dichloroisonicotinic acid; JA: Jasmonic acid; NAC: NAM ATAF1 and CUC2–like transcription factor; MEME: Multiple Em for motif elicitation; Myc2: Myelocytomatosis 2; Os: *Oryza sativa*; PR: Pathogenesis related; qRT-PCR: Quantitative reverse transcription-polymerase chain reaction; RD26: Response to dehydration 26; SA: Salicylic acid; SND: Secondary wall-associated *NAC Domain*; SWN: Swinger; Ta: *Triticum aestivum*; Zm: *Zea mays.*

## Competing interests

The authors declare that they have no competing interests.

## Authors’ contributions

AMV, HBD and LMV have conceptualized the research. SM and HBD have generated, isolated and characterized the mutants used in this study and performed the microarray analysis. AMV, SM, HBD and LMV have interpreted the microarray results. AMV has performed the experiments that have not yet been mentioned. AMV and LMV have interpreted the latter results and have written the manuscript. All authors read and approved the final manuscript.

## Supplementary Material

Additional file 1: Table S1 Chromosomal positions of the 116 identified ZmNAC genes in the maize genome. Click here for file

Additional file 2: Figure S1 Domain architecture of maize NAC proteins. A multiple alignment of the consensus sequences of the whole length NAC proteins from each clade was compiled using ClustalW 2.0. Amino acid residues present in at least 50% of the subclade members are displayed in the consensus sequences. Click here for file

Additional file 3: Table S2 A list of motifs detected within the 116 NAC proteins. The protein sequences were screened with MEME (Multiple Em for Motif Elicitation, http://meme.nbcr.net/meme/cgi-bin/meme.cgi ) at a cut off p-value of e^-10^. The motif location is given as follows: C-/N-term. – C-/N- terminus, NAC – NAC domain, sd. A-E – subdomain A-E. Click here for file
