# Supplementary material for: Two recently duplicated maize NAC transcription factor paralogs are induced in response to Colletotrichum graminicola infection
Source: BMC Plant Biol. 2013 May 29;13:85. doi: 10.1186/1471-2229-13-85 (PMC3694029; doi:10.1186/1471-2229-13-85)
Supplement: Additional file 2: Figure S1 — Domain architecture of maize NAC proteins. A multiple alignment of the consensus sequences of the whole length NAC proteins from each clade was compiled using ClustalW 2.0. Amino acid residues present in at least 50% of the subclade members are displayed in the consensus sequences. [file 1471-2229-13-85-S2.docx]

**Supplemental Figure S1**

**
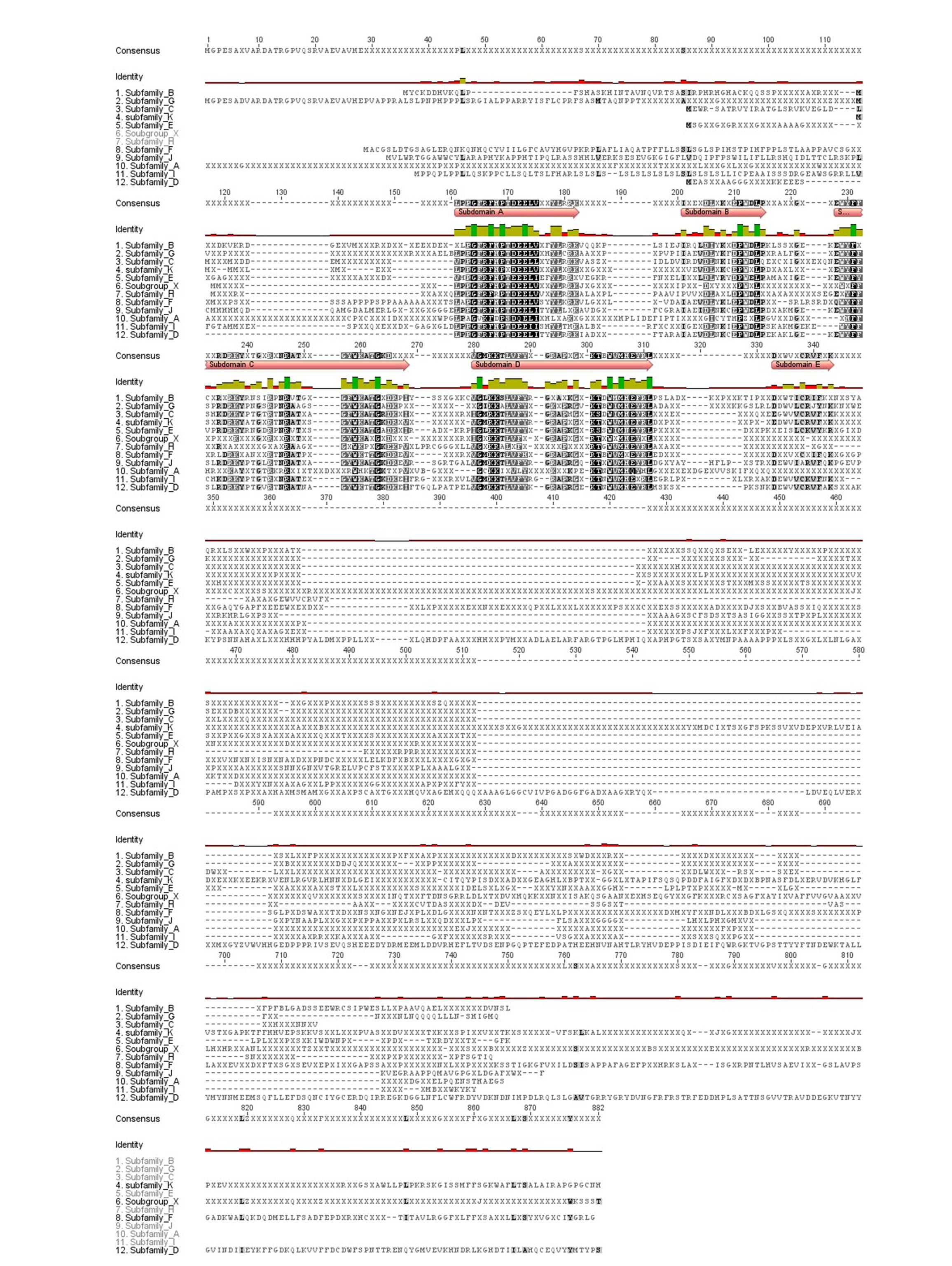
**

**Supplemental Figure S1.** Domain architecture of maize NAC proteins.

A multiple alignment of the consensus sequences of the whole length NAC proteins from each clade was compiled using ClustalW 2.0. Amino acid residues present in at least 50% of the subclade members are displayed in the consensus sequences.
