# Supplementary material for: Two recently duplicated maize NAC transcription factor paralogs are induced in response to Colletotrichum graminicola infection
Source: BMC Plant Biol. 2013 May 29;13:85. doi: 10.1186/1471-2229-13-85 (PMC3694029; doi:10.1186/1471-2229-13-85)
Supplement: Additional file 3: Table S2 — A list of motifs detected within the 116 NAC proteins. The protein sequences were screened with MEME (Multiple Em for Motif Elicitation, http://meme.nbcr.net/meme/cgi-bin/meme.cgi ) at a cut off p-value of e-10. The motif location is given as follows: C-/N-term. – C-/N- terminus, NAC – NAC domain, sd. A-E – subdomain A-E. [file 1471-2229-13-85-S3.docx]

**Supplemental Table S1**

**
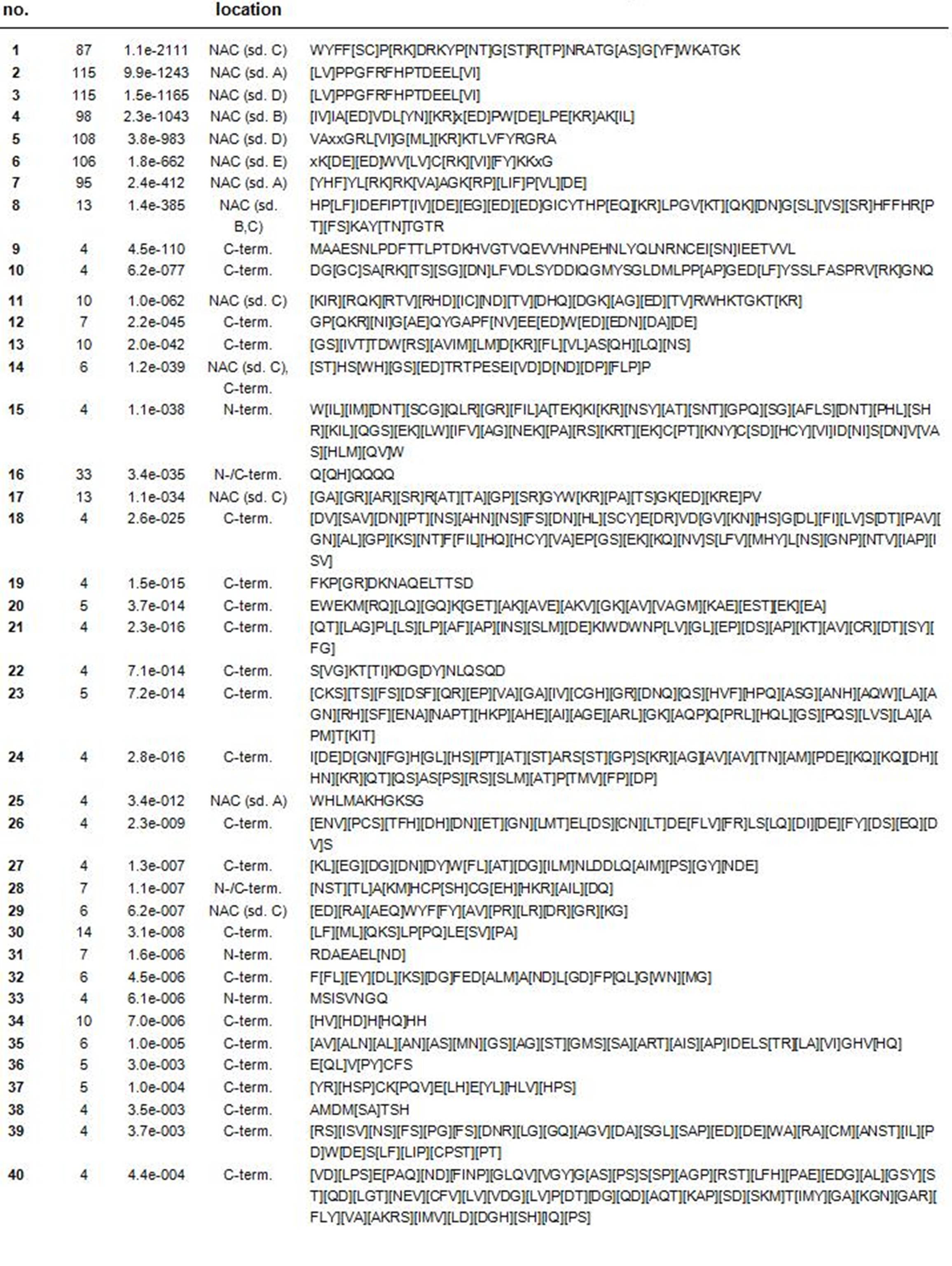
**

**Supplemental Table S1.** A list of motifs detected within the 116 NAC proteins.

The protein sequences were screened with MEME (Multiple Em for Motif Elicitation, <http://meme.nbcr.net/meme/cgi-bin/meme.cgi> ) at a cut off p-value of e^-10^. The motif location is given as follows: C-/N-term. – C-/N- terminus, NAC – NAC domain, sd. A-E – subdomain A-E.
